# Repurposing Napabucasin as an Antimicrobial Agent against Oral Streptococcal Biofilms

**DOI:** 10.1155/2020/8379526

**Published:** 2020-11-20

**Authors:** Xinyi Kuang, Tao Yang, Chenzi Zhang, Xian Peng, Yuan Ju, Chungen Li, Xuedong Zhou, Youfu Luo, Xin Xu

**Affiliations:** ^1^State Key Laboratory of Oral Diseases & National Clinical Research Center for Oral Diseases & Department of Cariology and Endodontics, West China Hospital of Stomatology, Sichuan University, Chengdu, China; ^2^Laboratory of Human Disease and Immunotherapies, West China Hospital, Sichuan University, Chengdu, China; ^3^State Key Laboratory of Biotherapy and Cancer Center, West China Hospital, West China Medical School, Sichuan University, Chengdu, China

## Abstract

**Objectives:**

Disruption of microbial biofilms is an effective way to control dental caries. Drug resistance and side effects of the existing antimicrobials necessitate the development of novel antibacterial agents. The current study was aimed at investigating the antibacterial activities of the repurposed natural compound napabucasin against oral streptococci.

**Methods:**

The minimum inhibitory concentration, minimum bactericidal concentration, minimum biofilm inhibition concentration, and minimum biofilm reduction concentration of *Streptococcus mutans*, *Streptococcus gordonii*, and *Streptococcus sanguinis* were examined by a microdilution method. Cytotoxicity of napabucasin against human oral keratinocytes, human gingival epithelia, and macrophage RAW264.7 was evaluated by CCK8 assays. The dead/live bacterium and exopolysaccharide in the napabucasin-treated multispecies biofilms were evaluated by confocal laser scanning microscopy. Microbial composition within the napabucasin-treated biofilms was further visualized by fluorescent in situ hybridization and qPCR. And the cariogenicity of napabucasin-treated biofilms was evaluated by transverse microradiography.

**Results:**

Napabucasin exhibited good antimicrobial activity against oral streptococcal planktonic cultures and biofilms but with lessened cytotoxicity as compared to chlorhexidine. Napabucasin reduced the cariogenic *S. mutans* and increased the proportion of the commensal *S. gordonii* in the multispecies biofilms. More importantly, napabucasin significantly reduced the demineralization capability of biofilms on tooth enamels.

**Conclusion:**

Napabucasin shows lessened cytotoxicity and comparable antimicrobial effects to chlorhexidine. Repurposing napabucasin may represent a promising adjuvant for the management of dental caries.

## 1. Introduction

Dental caries is one of the most prevalent diseases incurring large expenditures worldwide [1, 2]. It is a slowly progressive chronic disease initiated by oral biofilms and associated with multiple risk factors [3]. Cariogenic bacteria such as *Streptococcus mutans* dynamically compete with commensal bacteria including *Streptococcus sanguinis* and *Streptococcus gordonii* within the oral biofilm. Given the disequilibrium of the microbial ecology, the microbial metabolism of carbohydrates can lead to continuous decline of pH at the biofilm and tooth hard tissue interface, consequently causing demineralization of tooth hard tissue, and dental caries gradually occurs [3–7].

Mechanical plaque control is the mainstay for the control of oral biofilms and dental caries, but it heavily relies on individuals' compliance. To supplement mechanical plaque control, mouth rinses with antiplaque properties are well recommended [8]. Chlorhexidine (CHX) is one of the most common antimicrobial agents used as mouth rinse [9–11]. However, longtime usage of CHX could cause drug resistance and side effects such as taste confusions or tooth staining [11]. Therefore, alternative antimicrobial agents with comparable effectiveness but lessened side effects are needed for the better control of the oral biofilm [12].

**Figure 1:**
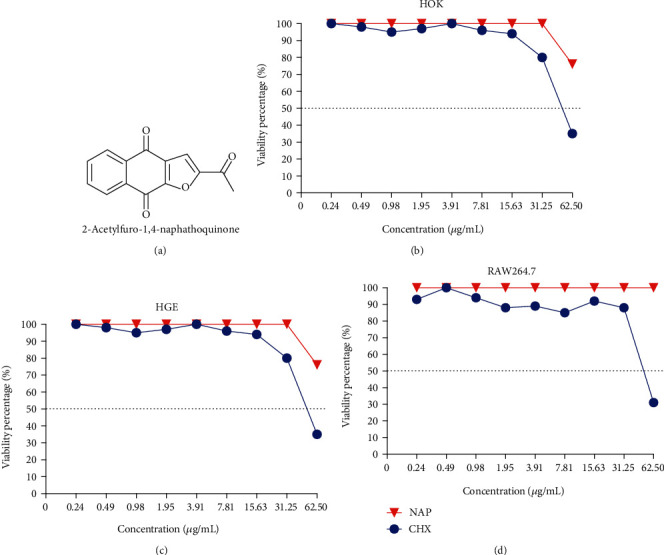
Cytotoxicity of NAP on human oral keratinocytes, human gingival epithelial cells, and macrophages. (a) Chemical structure of napabucasin; (b) viability of HOK treated with NAP and CHX: IC50_NAP_ > 62.5*μ*g/mL; IC50_CHX_ = 31.25 ~ 62.5*μ*g/mL; (c) viability of HGE treated with NAP and CHX: IC50_NAP_ > 62.5*μ*g/mL; IC50_CHX_ = 31.25 ~ 62.5*μ*g/mL; (d) viability of RAW264.7 treated with NAP and CHX: IC50_NAP_ > 62.5*μ*g/mL; IC50_CHX_ = 31.25 ~ 62.5*μ*g/mL. HOK: human oral keratinocytes; HGE: human gingival epithelial cells; RAW264.7: macrophage RAW264.7.

Drug repurposing has garnered increasing attention as an alternative strategy to identify new antimicrobial agents for its efficiency in reducing time, cost, and risks associated with the development of novel antibiotics [13, 14]. In an effort to repurpose existing drugs as antibacterial agents, we have recently screened from a library of bioactive molecules against *Streptococcus mutans* and identified the natural compound napabucasin (NAP) (Figure 1(a)), namely, 2-acetylfuro-1,4-naphathoquinone. 2-Acetylfuro-1,4-naphathoquinone is one of the chemical constituents first isolated from *Newbouldia laevi*s [15]*. N. laevis* is widely used in the African folk medicine and has been reported to reduce dental caries and other diseases [16]. Previous studies reported the antibacterial activity of 2-acetylfuro-1,4-naphathoquinone against *Escherichia coli*, *Streptococcus faecalis*, and *Staphylococcus aureus* [15, 17]. A recent study also showed its antimycobacterial activity for the treatment of tuberculosis [18]. In addition, the NAP is in phase III clinical trials for the treatment of cancers (i.e., gastric cancer, pancreatic cancer, and colorectal cancer) [19–23]. However, there is no data to support its activity against oral pathogens.

The purpose of this study is to investigate the antimicrobial activity of NAP against oral streptococci.

## 2. Materials and Methods

### 2.1. Test Bacteria and Chemicals

*Streptococcus mutans* UA159, *Streptococcus gordonii* DL1, and *Streptococcus sanguinis* ATCC 10556 were kindly provided by the State Key Laboratory of Oral Diseases (Sichuan University, Chengdu, China). *S. mutans*, *S. gordonii*, and *S. sanguinis* were routinely grown at 37°C under aerobic condition (5% CO_2_) in brain heart infusion broth (BHI; Difco, Sparks, MD). Inoculum for the experiment was adjusted to 1 × 10^8^CFU/mL for *S. mutans*, *S. gordonii*, and *S. sanguinis* based on the OD_600 nm_ versus CFU/mL graph of each bacterium and further 1 : 100 diluted in the growth culture. When needed, medium was supplemented with 1% sucrose (designated BHIS).

Napabucasin was purchased from Bide Pharmatech Ltd. and prepared in DMSO at a stocking concentration of 100 mg/mL.

### 2.2. Susceptibility Tests

#### 2.2.1. Bacterial Susceptibility Test

The minimum inhibitory concentration (MIC) and minimum bactericidal concentration (MBC) of NAP against *S. mutans*, *S. gordonii*, and *S. sanguinis* were determined by a microdilution method in BHI, as described previously [24, 25]. The concentrations of NAP ranged from 0.97 to 1000 *μ*g/mL (twofold dilutions). BHI broth containing equivalent DMSO (1% to 0.001%, *v/v*) was used as a solvent control and ran simultaneously to control for the possible growth inhibition caused by the added DMSO. CHX was used as a positive control, cell control (test bacteria and BHI broth) was used as a negative control, and BHI broth was used as a blank control.

#### 2.2.2. Biofilm Susceptibility Test

The minimum biofilm inhibition concentration (MBIC) was used to evaluate the effect of NAP on biofilm formation [26]. *S. mutans*, *S. gordonii*, or *S. sanguinis* (1 × 10^7^CFU/mL, 10 *μ*L/well) were grown in BHIS with twofold serial dilution of NAP (200 *μ*L/well) ranging from 0.12 to 125 *μ*g/mL at 37°C for 24 h. A parallel study was also performed with BHIS as a negative control. Then, the supernatants from the wells were decanted, and the adherent biofilm was washed three times with PBS to remove the planktonic cells. Fixed with methanol for 15 min and air-dried at room temperature, the biofilm was stained with 0.1% (wt/vol) crystal violet (Sigma) for 5 min, rinsed with deionized water until blank control wells were colorless, and added 200 *μ*L of 95% ethanol to each crystal violet-stained well. Subsequently, the plate was rocked 30 min at room temperature, and the absorbance at 595 nm was recorded. The percentage of inhibition was calculated using the equation: (*A*_595_ofnegativecontrol − *A*_595_ofthetestgroup)/A_595_ofnegativecontrol × 100%. The MBIC was defined as the lowest agent concentration that showed 90% or more inhibition of biofilm formation.

The effect of NAP on the 1-day-developed biofilm was examined by the minimum biofilm reduction concentration (MBRC). *S. mutans*, *S. gordonii*, or *S. sanguinis* (1 × 10^7^CFU/mL, 200 *μ*L/well) in BHIS was added to a 96-well polystyrene tissue culture plate. After anaerobic incubation at 37°C for 24 h, the supernatants were removed and washed with PBS three times without disrupting the integrity of biofilms. Fresh BHIS containing NAP ranging from 0.12 to 125 *μ*g/mL were added to each well and incubated at 37°C for 24 h. The negative control was biofilms in BHIS without NAP. The biofilm was fixed, air-dried, stained, and quantified as described above. The MBRC was defined as the lowest agent concentration that showed 90% or more reduction of the biofilm.

### 2.3. In Vitro Cytotoxicity/Viability Assay

Cell viability was evaluated by using the Cell Counting Kit-8 (CCK-8, Dojindo, Kumamoto, Japan) assay as described by Park et al. [27]. Test cells were human oral keratinocytes (HOK), human gingival epithelia (HGE), and macrophage RAW264.7 (RAW264.7). Cells were plated in 96-well plates at 10,000 cells/well in minimum essential medium with the Dulbecco's modified Eagle's medium (DMEM) supplemented with 10% fetal bovine serum and 1% antibiotic-antimycotic. The cells were grown in a humidified environment with 5% CO_2_ at 37°C for 24 hours. Considering the short exposure duration of oral cells to mouth rinses, cells were treated with medium containing NAP (0.12 to 62.5 *μ*g/mL) and positive control CHX for 5 min [27–29]. Then, the cells were washed with PBS twice and were added with the fresh medium (200 *μ*L/well). Each well was added with 10 *μ*L of CCK-8, and after incubation in the CO_2_ incubator for 1 h to 1.5 h, absorbance was measured at the wavelength of 450 nm. The cell viability was calculated according to the following formula: (%) = (*A*_450_oftestgroup − *A*_450_ofblankcontrol)/(*A*_450_ofnegativecontrol − *A*_450_ofblankcontrol) × 100%.

### 2.4. Multispecies Biofilm Imaging

The multispecies biofilms were cultivated in accordance with a previous study [30]. Overnight cultures of *S. mutans*, *S. gordonii*, and *S. sanguinis* were simultaneously inoculated (inoculum ratio = 1 : 1 : 1). The chemotaxis chamber *μ*-Slide, which has extremely low values of birefringence and autofluorescence, was used for bacterial culture and confocal microscopy [31]. Bacterial suspensions (1 × 10^5^CFU/mL for each strain) were mixed in 300 *μ*L BHI containing 1% sucrose (BHIS) in the *μ*-Slide (8 wells, 80826, Ibidi) at 37°C for 24 h. Then, biofilms were exposed to PBS, 62.5 *μ*g/mL NAP, and 0.2% CHX for 3 days (5 min, three times per day). This short-term repeated treatment was to simulate the daily exposure to the mouth rinses [32].

For dead/live imaging, biofilms were stained with fluorescent LIVE/DEAD BacLight Bacterial Viability stain (Molecular Probes, Invitrogen) containing SYTO 9 and propidium iodide according to the manufacturer's instructions. The labeled biofilms were imaged with a DMIRE2 confocal laser scanning microscope (Leica, Wetzlar, Germany) equipped with a 60x oil immersion objective lens.

For extracellular polysaccharide (EPS) staining, the bacterial cell and the EPS were stained with SYTO 9 (Molecular Probes) and Alexa Fluor 647-labeled dextran conjugate (Molecular Probes) as described previously [33]. The biofilms were captured with a Leica DMIRE2 confocal laser scanning microscope as in live/dead imaging.

For fluorescent *in situ* hybridization imaging, biofilms were fixed in 4% paraformaldehyde overnight and investigated by species-specific probes [34]. The multispecies biofilms were imaged with a confocal laser scanning microscope (FV1000, Olympus, Tokyo, Japan).

All three-dimensional reconstructions of the biofilms were performed with Imaris 7.0.0 (Bitplane, Zürich, Switzerland). The quantification of the dead/live and EPS/bacteria volume ratio was, respectively, performed with Image-Pro Plus (Media Cybernetics, Silver Spring, MD, USA) and COMSTAT (http://www.image-analysis.dk) [33].

### 2.5. DNA Isolation and Real-Time PCR

Total DNA of biofilms were isolated and purified using a TIANamp Bacteria DNA kit (TIANGEN, Beijing, China). The purity and concentration of DNA were detected with a NanoDrop 2000 spectrophotometer (Thermo Scientific, Waltham, MA, USA). The extracts were stored at -20°C until use. TaqMan real-time polymerase chain reaction (Life Technologies, Carlsbad, CA, USA) was used to quantify the absolute number of *S. mutans*, *S. gordonii*, and *S. sanguinis* as described by the manufacturer (Takara, Dalian, China).

### 2.6. Transverse Microradiography

Human teeth free of white spots, cracks, and other defects that had been extracted for periodontal or impacted reasons were collected under a protocol approved by the Ethics Committee of West China Hospital of Stomatology, Sichuan University (WCHSIRB-D-2018-107). Crowns were separated from roots and cut into four sections measuring 5mm × 5mm × 2mm by using a diamond-coated saw (Struers Minitom; Struers, Copenhagen, Denmark) under continuous water cooling. The enamel slabs were embedded in polymethylmethacrylate and painted with two layers of acid-resistant nail varnish, leaving a 4mm × 4mm window of the exposed enamel surface. Then, these surfaces were polished progressively with waterproof silicon carbide abrasive papers (800–4000 grit; Struers, Copenhagen, Denmark) and were ultrasonically cleaned in a deionized water for 5 min to remove the residual abrasives. All slabs were sterilized with ethylene oxide [35]. The 30 specimens were randomly divided into 3 groups: PBS, 62.5 *μ*g/mL NAP, and 0.2% CHX. Enamel slabs obtained from the same tooth were evenly distributed to each test group.

Overnight cultures of *S. mutans*, *S. gordonii*, and *S. sanguinis* (1 × 10^7^CFU/mL) were simultaneously inoculated on enamel slabs in a 24-well cell culture plate with BHIS at 37°C. Slabs with biofilms were then exposed to PBS, 62.5 *μ*g/mL NAP, and 0.2% CHX for 5 days (5 min, three times per day). The 5-day demineralization duration was in accordance with previous studies [36, 37]. All specimens were then washed in PBS and refreshed with BHIS after every exposure. After 5 days of treatment, specimens were taken out and rinsed with PBS to remove the biofilms. Then, specimens were cut again and polished with a hand plane—parallel from both sides with waterproof silicon carbide abrasive papers (800–4000 grit; Struers, Copenhagen, Denmark) to thickness ranging around 150 nm [38]. X-ray films of experimental lesions were acquired with an X-ray generator (Softex, Japan) equipped with a microradiography camera and then were further examined using a Zeiss AXIO Imager A2 microscope (Carl Zeiss, Germany). Quantitative data was acquired by a calibrated analysis system TMR2006 (Inspektor Research Systems BV, Netherlands) [39]. Data are obtained as the mean of 10 separate samples.

### 2.7. Statistical Analysis

All experiments were repeated at least three times independently. One-way analyses of variance (ANOVAs) and the Student-Newman-Keuls test were used to compare differences. Differences were considered significant when *P* < 0.05. Statistical analyses were performed with the SPSS software, version 16.0 (SPSS Inc., Chicago, IL, USA).

## 3. Results

### 3.1. NAP Exhibits Good Antimicrobial Activity against Oral Streptococci

NAP inhibited the planktonic growth of *S. mutans*, *S. gordonii*, and *S. sanguinis* with minimum inhibitory concentration (MIC) ranging from 0.49 *μ*g/mL to 3.91 *μ*g/mL and minimum bactericidal concentration (MBC) ranging from 0.98 *μ*g/mL to 15.63 *μ*g/mL. Besides, NAP inhibited the biofilms of *S. mutans*, *S. gordonii*, and *S. sanguinis* with minimum biofilm inhibitory concentration (MBIC) ranging from 0.49 *μ*g/mL to 1.95 *μ*g/mL and minimum biofilm reduction concentration (MBRC) ranging from 3.91 *μ*g/mL to 62.5 *μ*g/mL (Table 1). CHX, as a positive control, inhibited the planktonic growth and biofilms of *S. mutans*, *S. gordonii*, and *S. sanguinis* with MIC ranging from 0.49 *μ*g/mL to 1.95 *μ*g/mL, MBC ranging from 3.91 *μ*g/mL to 7.81 *μ*g/mL, MBIC ranging from 0.98 *μ*g/mL to 3.91 *μ*g/mL, and MBRC ranging from 3.91 *μ*g/mL to 62.5 *μ*g/mL (Table 1).

### 3.2. NAP Shows Lessened Cytotoxicity against Human Oral Cells Relative to Chlorhexidine

The cytotoxicity of NAP against human oral keratinocytes (HOK), human gingival epithelia (HGE), and macrophage RAW264.7 (RAW264.7) was evaluated by measuring the cell viability after drug exposure duration of 5 min. NAP showed lessened cytotoxicity against HOK, HGE, and RAW264.7 compared with CHX. More importantly, the IC50 of NAP on HOK, HGE, and RAW264.7 (IC50 > 62.5*μ*g/mL) were higher than its minimal biofilm reduction concentrations against an oral streptococcal biofilm (MBRC ranging from 3.91 *μ*g/mL to 62.5 *μ*g/mL), suggesting that NAP is safe for use as an antimicrobial agent at the exposure duration (Figures 1(b)–1(d)).

### 3.3. NAP Inhibits the Development of Multispecies Biofilms

**Figure 2:**
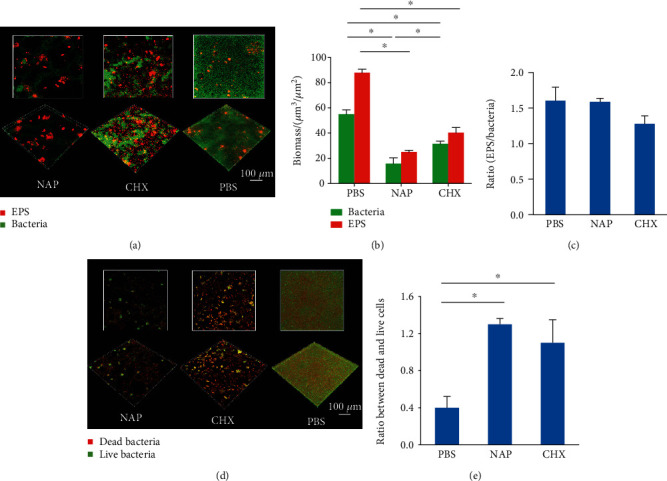
The antimicrobial effects of NAP against oral streptococcal multispecies biofilms. (a) Representative image of multispecies biofilms treated with NAP. Bacteria stained green; extracellular polysaccharides (EPS) stained red. (b) The volume of EPS and bacteria within the biofilms; (c) the ratio of EPS/bacteria within the biofilms; (d) representative image of dead/live bacteria within the multispecies biofilms after treatment; live bacteria stained green; dead bacteria stained red; (e) quantitative ratio of dead and live bacteria after treatment. Data are presented as mean ± standarddeviation (SD). ^∗^*P* < 0.05.

**Figure 3:**
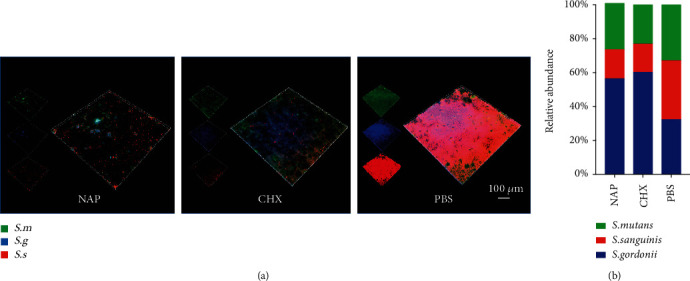
Effects of NAP on the composition shift of multispecies biofilms. (a) Representative fluorescent in situ hybridization (FISH) images of multispecies biofilms treated with NAP, CHX, and phosphate-buffered saline (PBS). *S. mutans* (*S. m*, green), *S. gordonii* (*S. g*, blue), and *S. sanguinis* (*S. s*, red) were labeled with species-specific FISH probes. Images were captured with a fluorescence microscope at 60x magnification. (b) The ratio of *S. mutans*, *S. gordonii*, and *S. sanguinis* in multispecies biofilms quantified by qPCR. ^∗^*P* < 0.05.

The antimicrobial effects of NAP were further evaluated with multispecies biofilms. Both NAP and CHX treatment disrupted the structural integrity of multispecies biofilms significantly (Figure 2(a)). NAP treatment further reduced the bacteria within the oral streptococcal biofilms as compared to CHX (Figure 2(b)). In addition, the biofilms treated with NAP showed an equivalent dead/live cell ratio as well as an EPS/bacteria ratio as compared to the CHX-treated ones (Figures 2(c)–2(e)). Fluorescent *in situ* hybridization imaging and qPCR were conducted to evaluate the effect of NAP on multispecies biofilm composition. As shown in Figures 3(a) and 3(b), both NAP and CHX treatments significantly reduced the total amount of streptococci. NAP inhibited *S. mutans* and S. *sanguinis* but increased the proportion of the commensal *S. gordonii* within the multispecies biofilms.

### 3.4. NAP Reduces the Demineralization Capability of Streptococcal Biofilms on Enamel

**Figure 4:**
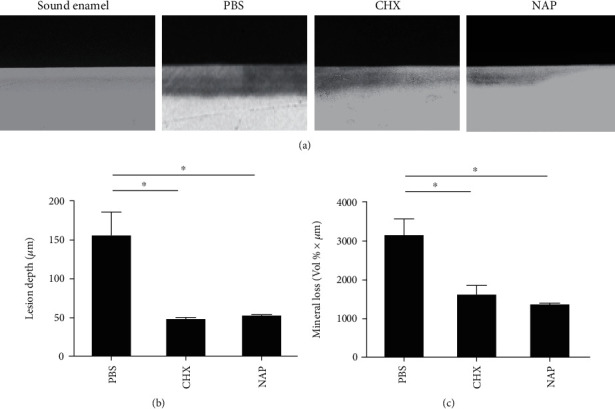
The antidemineralization effect of NAP against multispecies biofilms. (a) Representative transverse microradiography (TMR) images of human enamel discs exposed to 5-day biofilm-induced experimental demineralization. The high-density regions represent the sound enamel tissues, while the low-density shadows indicate the caries-like lesions. (b) Lesion depth and (c) mineral loss were calculated. Data are presented as mean ± SD. ^∗^*P* < 0.05.

We further evaluated the inhibitory effects of NAP on the cariogenicity of oral biofilms by quantifying biofilm-induced demineralization on human enamel slabs. As shown by the transverse microradiography data, the depth of a biofilm-induced lesion and the mineral loss of enamel were significantly reduced when treated with CHX and NAP compared to the negative control. NAP and CHX showed a comparable inhibitory effect on the biofilm-induced demineralization (Figure 4).

## 4. Discussion

As a biofilm-associated chronic disease, effective biofilm control is critical for dental caries management. Antimicrobial agents, as supplements to insufficient mechanical removal, have been used to control oral biofilms for years. Clinical trials have shown that long-time usage of antimicrobial mouth rinses can significantly reduce *S. mutans* in saliva [40, 41] and reduce the incidence of caries among people at high caries risk [42, 43]. However, CHX had only a superficial bactericidal effect on dental plaque and exhibited noticeable cytotoxicity as dose increases [44, 45]. Long-term repeated exposure to CHX could induce drug resistance in oral microbes such as *S. gordonii*, *Enterococcus faecalis*, *Fusobacterium nucleatum*, and *Porphyromonas gingivalis* [46, 47]. In addition, CHX can cause tooth or tongue staining and taste confusions in patients [48]. The adversary effects of CHX necessitate the development of novel agents to control oral biofilms. Here, we demonstrated that the natural compound NAP possessed comparable antimicrobial activity with CHX against oral streptococcal biofilms but with relatively lower cytotoxicity, representing a promising novel agent in the control of dental caries. NAP is also an anticancer drug that is in phase III clinical trials for cancer treatment. Drug repurposing is an effective drug development strategy [49]. The repurposed use of the anticancer drug toremifene showed good antimicrobial activity against oral pathogens *P. gingivalis* and *S. mutans* by damaging the bacterial membrane [50]. The antiasthma drug zafirlukast also showed potent antimicrobial activity against *P. gingivalis* and *S. mutans* [51]. The repurposed use of NAP in the current study also showed potent antimicrobial activity against oral streptococci in either planktonic culture or biofilm.

Oral biofilms are microbes embedded within an EPS matrix that functions as a “glue” to form a cohesive and adherent ecosystem. EPS is well recognized as a critical cariogenic factor of a streptococcal biofilm [5, 33, 52–54]. Disruption of EPS can disperse the biofilm and increase the sensitivity of bacteria to antibiotics [54, 55]. Previous studies verified that proanthocyanidins (PACs) in cranberry could reduce the amount of EPS, break down microarchitecture of the cariogenic biofilm, and reduce the incidence of smooth-surface caries in rats [56, 57]. The current study found that NAP could significantly inhibit EPS production in the biofilm and thus disrupted the integrity of the streptococcal biofilm, further supporting its potential use as a plaque control measure that could supplement the management of dental caries.

The cariogenicity of the oral biofilm is closely associated with microbial interactions between cariogenic *S. mutans* and commensal streptococci such as *S. sanguinis* and *S. gordonii*. Disequilibrium within the plaque biofilms is the initiating event that mediates the transition from health to disease [58]. Changes in host diet such as excessive carbohydrate consumption promote accumulation of acid-producing organisms that trigger ecological alteration towards cariogenic microbiota [5]. The most typical acid-producing organism, *S. mutans*, can drive the dysbiosis of the oral microbial ecology and ultimately lead to the occurrence of dental caries [59, 60]. Inhibiting *S. mutans* with increasing relative abundance of commensal streptococci is believed to be “ecologically safe” for the control of oral biofilms [6, 24, 53, 61]. Our previous study showed that the combinatory use of arginine and NaF could inhibit *S. mutans* but enrich the commensal *S. sanguinis* in the multispecies biofilms, representing an ecological approach to the management of dental caries [6]. In this study, multispecies biofilms consisting of *S. mutans*, *S. gordonii*, and *S. sanguinis* were established and we found that NAP could suppress cariogenic *S. mutans* but increase the proportion of *S. gordonii* within the multispecies biofilms. More importantly, NAP treatment could significantly halt the biofilm-induced demineralization of tooth enamel, suggesting that NAP could be a good candidate for daily use mouth rinse.

Biocompatibility is a critical factor for daily use mouth rinse. Based on the clinical data reported in the previous phase I and II study, patients with advanced malignancies received napabucasin orally at a dose of 240 mg twice a day. Adverse events were generally mild and predominantly included diarrhea, abdominal pain, nausea, and fatigue [22, 62]. The current study further evaluated the cytotoxicity of NAP on human oral cells such as HOK, HGE, and macrophage RAW264.7. Lower cytotoxicity of NAP against all these cells was observed as compared to that of CHX. These data further support repurposing NAP as an antimicrobial compound that can be tropically used for the control of oral biofilms.

## 5. Conclusion

In summary, this study for the first time demonstrated that NAP exhibited good antibacterial capability against oral streptococcal biofilms with lower cytotoxicity. NAP can disperse the multispecies biofilms and reduce the biofilm-induced demineralization of tooth hard tissue with decreasing relative abundance of *S. mutans* in the biofilms. Further *in vivo* studies are still needed to translate this promising repurposed natural compound to the management of dental caries.

## Figures and Tables

**Table 1:** MICs, MBCs, MBICs, and MBRCs of NAP and CHX against *S. mutans*, *S. gordonii*, and *S. sanguinis* strains.

Bacterial strain (*μ*g/mL)	Planktonic cells				Biofilm			
	MIC		MBC		MBIC		MBRC	
	NAP	CHX	NAP	CHX	NAP	CHX	NAP	CHX
*S. mutans*	3.91	1.95	15.63	7.81	1.95	0.98	62.50	62.50
*S. gordonii*	0.49	3.91	0.98	7.81	0.49	3.91	15.63	15.63
*S. sanguinis*	0.49	0.49	15.63	3.91	1.95	1.95	3.91	3.91

MIC: minimum inhibitory concentration; MBC: minimum bactericidal concentration; MBIC: minimum biofilm inhibition concentrations; MBRC: minimum biofilm reduction concentrations.

## Data Availability

All data used during the study are available in the article and can be solicited from the corresponding author.
