# Corrigendum to “Repurposing Napabucasin as an Antimicrobial Agent against Oral Streptococcal Biofilms”

**DOI:** 10.1155/2021/9897654

**Published:** 2021-12-11

**Authors:** Xinyi Kuang, Tao Yang, Chenzi Zhang, Xian Peng, Yuan Ju, Chungen Li, Xuedong Zhou, Youfu Luo, Xin Xu

**Affiliations:** ^1^State Key Laboratory of Oral Diseases & National Clinical Research Center for Oral Diseases & Department of Cariology and Endodontics, West China Hospital of Stomatology, Sichuan University, Chengdu, China; ^2^Laboratory of Human Disease and Immunotherapies, West China Hospital, Sichuan University, Chengdu, China; ^3^State Key Laboratory of Biotherapy and Cancer Center, West China Hospital, West China Medical School, Sichuan University, Chengdu, China

In the article titled “Repurposing Napabucasin as an Antimicrobial Agent against Oral Streptococcal Biofilms” [1], the authors mistakenly generated the line graph of Figure 1(c) using the data from Figure 1(b). The corrected figure is provided as follows.

## Figures and Tables

**Figure 1:**
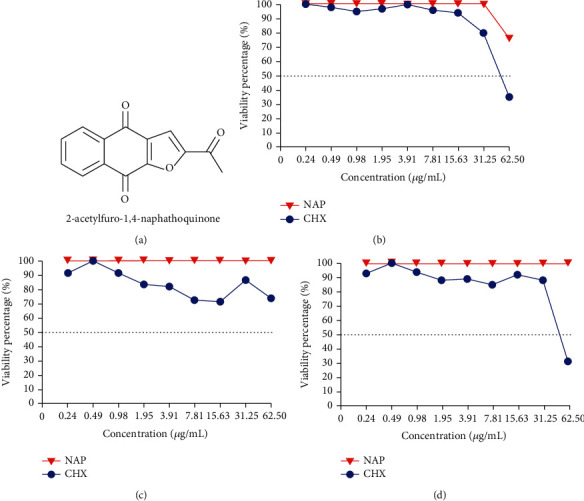
Cytotoxicity of NAP on human oral keratinocytes, human gingival epithelial cells, and macrophages. (a) Chemical structure of napabucasin; (b) viability of HOK treated with NAP and CHX: IC50_NAP_ > 62.5 *μ*g/mL and IC50_CHX_ = 31.25 ~ 62.5 *μ*g/mL; (c) viability of HGE treated with NAP and CHX: IC50_NAP_ > 62.5 *μ*g/mL and IC50_CHX_ > 62.5 *μ*g/mL; (d) viability of RAW264.7 treated with NAP and CHX: IC50_NAP_ > 62.5 *μ*g/mL and IC50_CHX_ = 31.25 ~ 62.5 *μ*g/mL. HOK: human oral keratinocytes; HGE: human gingival epithelial cells; RAW264.7: macrophages RAW264.7.
